# Potential Savings from Visit Reduction of Continuous Intraocular Pressure Monitoring

**DOI:** 10.5005/jp-journals-10008-1246

**Published:** 2018-08-01

**Authors:** Jiaxi Dong, Zeba A Syed, Kenneth Fan, Ali F Yahya, Samir A Melki

**Affiliations:** 1 Student, Boston Eye Group, Boston, Massachusetts, USA; 2 Physician, Cornea and Refractive Surgery Service, Massachusetts Eye and Ear Infirmary, Boston, Massachusetts, USA

**Keywords:** Cost-effectiveness of Glaucoma Management, Economics of glaucoma, Glaucoma diagnostics, IOP monitoring

## Abstract

**Introduction:**

A continuous method of measuring intraocular pressures (IOP) could be advantageous in the management of glaucoma. This report aims to analyze the potential savings from visit reduction of continuous IOP measurements obtained with an intraocular device.

**Materials and methods:**

We constructed a model adapted from a prior study based on the number of glaucoma patients among 5% of the Medicare population.

**Results:**

We found that the implementation of a device that continuously measures IOP can result in a reduction of 23.21% in yearly costs from glaucoma outpatient visits.

**Conclusion:**

Continuous IOP monitoring has the potential to alleviate the economic burden of the current management structure of patients with glaucoma.

**Clinical Significance:**

In an era of elevated healthcare costs, continuous IOP monitoring offers an option to improve the care of glaucoma patients through visit reduction, also resulting in a 23.21% reduction in yearly expenses related to glaucoma clinical visits.

**How to cite this article:** Dong J, Syed ZA, Fan K, Yahya AF, Melki SA. Potential Savings from Visit Reduction of Continuous Intraocular Pressure Monitoring. J Curr Glaucoma Pract 2018;12(2):59-63.

## INTRODUCTION

Nearly three million Americans were afflicted with glaucoma in 2014, and this statistic is predicted to rise to four million by 2032.^1^ Glaucoma is the second most prevalent cause of vision loss in the United States of America, and its management inflicts great financial burden, accounting for 2.86 billion dollars in 2006.^2^

Lowering IOP, either medically, with a laser, or surgically, may slow or stop the advancement of glaucoma in early and late stages.^3^ In addition to elevated IOP, increased fluctuation of IOP is a possible risk factor for glaucoma progression. A greater than or equal to 2 mm Hg standard deviation (SD) in IOP variability is linked with 30% of visual field progression in open-angle glaucoma and 28.6% in angle closure glaucoma, as compared to 9.7% and 10% respectively in cases where the SD is under 2 mm Hg.^4^ However, other studies show mixed associations between IOP fluctuation and progression.

Goldmann applanation tonometry, the gold standard for IOP measurement, has an important weakness in that it offers a single and static IOP value. Such a measurement performed within normal office hours has less than a 25% chance of representing a patient’s peak IOP.^4^ Liu et al. found that peak IOP in most individuals occurs at night.^5^ Home tonometry devices offer an alternative, but these are limited by their expense, dependence on proper technique/positioning, and inability to measure IOP while asleep. As such, a reliable technique to continuously monitor IOP could be beneficial.

Recently, the ocular telemetry sensor (OTS) emerged as a possible means for continuous IOP measurement. The OTS is a contact lens that uses changes in the curvature of the cornea to extrapolate IOP variation. The data is collected by an external antenna mounted adjacent to the eye. Limitations of the OTS include patient pain and blurry vision.^6,7^

The implantable Wireless IOP Transducer (WIT aka ARGOS and EyeMate; Implandata ophthalmic products GmbH, Hannover, Germany) allows continuous IOP measurements. The device is an application-specific integrated circuit (ASIC) that merges the abilities of a pressure-detector, temperature-detector, identification encoder, analog-to-digital converter, and telemetry. The ASIC chip connects to a micro antenna. The OTS and WIT differ as the WIT measures the actual intraocular pressure, rather than of deriving it from corneal curvature.^8^ The device is powered through an external magnetic field generator and therefore does not necessitate batteries. The reader device receives electronic data transferred by the ASIC chip and can store at least 3,000 IOP readings.^9^ It also permits data collection on a continuous basis while the OTS can only measure IOP over a single 24-hour span.

In 2011, Todani et al. established WIT biocompatibi-lity in rabbit eyes for up to 25 months and demonstrated excellent IOP measurement reproducibility.^8^ The measurements obtained by the WIT were less variable than those taken by a pneumotonometer or Tonopen, and the authors reported SD of 0.81 mm Hg as compared to 2.70 mm Hg and 3.35 mm Hg respectively.^8^ In 2014, Melki et al. pioneered the use of WIT in a human eye.^9^ The device was biocompatible and provided reliable IOP measurements.^9^ A prospective, single-center clinical trial with one year of follow-up among six patients demonstrated that despite early postoperative anterior chamber inflammation among some eyes, the IOP sensor was tolerated well by all patients.^10^

The goal of this report is to analyze potential financial savings that could be achieved through visit reduction if continuous IOP measurements are reliably obtained with an intraocular device.

## MATERIALS AND METHODS

### Eligible Patients

We constructed a model based on the number of glaucoma patients among 5% of the medicare population as approached by Quigley et al.^11^ Because the system may only be implanted in glaucoma patients with prior or concurrent cataract surgery, we estimated the number of eligible patients by identifying the prevalence of cataracts in America (17.11%) and multiplying it by the figure for glaucoma patients recorded by the medicare study (163,972).^11,12^ This assumes that cataract and glaucoma occur independently of each other, which evidence supports.^13^

### Glaucoma Cost per Patient

The recommended number of visits among glaucoma patients is generally two to four annually.^14^ The frequency of outpatient appointments in advanced cases can be nearly twelve annually, but such unusual cases were not reflected in our model. Patients were distributed into one of three groups: mild glaucoma/glaucoma suspect, intermediate glaucoma, and severe glaucoma. Mild glaucoma/glaucoma suspect was defined as having minimal narrowing of the neuroretinal rim associated with limited damage on visual field testing (mean deviation less than -6 dB). Intermediate glaucoma included moderate narrowing of the rim associated with large visual fields defects that do not include fixation (mean deviation between -6 and -12 dB). Severe glaucoma had complete absence of the neuroretinal rim in at least three quadrants, with advanced visual field loss in both hemi-fields and possible involvement of fixation (mean deviation worse than -12 dB).^14^ The number of visits annually was assigned as two, three, and four for these patients respectively, as described previously.^14^ For the purposes of this model, we assumed that with the implantation of a continuous IOP monitoring device, all three categories could be assessed in a single comprehensive annual exam.

### Direct Savings

A statistical analysis was conducted on the direct savings of the device due to outpatient visit reduction. For each increase of 20% penetrance of the device, annual savings were calculated. This analysis was performed independently for the mild, intermediate, and severe glaucoma groups, as they each had differing appointment frequencies.

## RESULTS

### Visits and Costs

As per the results from the investigation of 1.38 million medicare beneficiaries, 163,972 made one or more claim under a glaucoma category in 2009.^11^ These patients generated 302,019 visits under various billing codes, from the IOP check (99212) to the more comprehensive evaluation (92014). Overall, the seven most common outpatient visit categories resulted in a yearly cost of $16,732,971.^11^ This figure only reflects outpatient visits. Among office visits, diagnostic tests, and surgical/laser procedures, office visits represent 46.3% of total spending.^11^ Spending on medications, for example, were not included. Among the listed visit types, the most common three types were 92012, 92014, and 99213 (contributing to 37.1% of total costs).

### Eligible Patients

A prerequisite for implantation of the system is prior or concurrent cataract surgery. If we assume that the risks of glaucoma and cataract are independent,^13^ the prevalence of cataract in the national and glaucoma populations should match. Per the National Eye Institute, the prevalence of cataract in America is 17.11%, and this figure increases with age.^12^ Hence, possible candidates in the 5% Medicare population would be 17.11% of 163,972 patients or 28,056 individuals.

### Patient Visits

Our review with glaucoma specialists revealed that patients receive a comprehensive exam coded as 92014 or 99214. Within our study, the price for 92014 ($70.84) is employed because it is utilized more commonly than 99214. Individuals with mild glaucoma have an additional outpatient visit every year (99213), and intermediate patients require a further brief visit (99212). Individuals with severe glaucoma are typically advised to see their ophthalmologist quarterly, with varying visit types. For our investigation, we assumed that patients with severe glaucoma receive one comprehensive, two intermediate, and one brief visit annually. This amounts to $113.71, $139.97 and $190.28 annual office visit cost for mild, intermediate, and severe patients respectively (Table 1).

Once a continuous IOP monitoring device has been implanted, we predict that individuals would visit their glaucoma specialist once annually if no surgical interventions are needed. Here, individuals are billed under the comprehensive 92014 ($70.84) code, because this single outpatient appointment likely involves studies including perimetry and optic nerve analysis. Hence, all patients’ yearly outpatient costs would be minimized to $70.84. The annual cost savings through decreased outpatient visits would be $42.87, $69.13, and $119.44 for each of the mild, intermediate, and severe glaucoma patients respectively (Table 1).

### Direct Savings

Because no data is available on the severity distribution of glaucoma cases in the United States, a model was created to allow for maximal flexibility. The model calculated three distinct sets of savings induced by the device, each assuming that 100% of the 28,056 eligible patients are part of one of the three severity categories (Table 2). Subsequently, the direct saving by the device from visit frequency reduction could be deducted from this table depending on penetrance (defined as patients who underwent device implantation and in whom the implant reduced clinical outpatient visits).

### Hypothetical Simulation

To illustrate the methodology of the model, we constructed a hypothetical scenario. We assumed that of the candidates from the 5% of Medicare population (28,056), 25% suffer from mild glaucoma, 50% suffer from intermediate glaucoma, and 25% suffer from severe glaucoma. This breakdown is an assumption, as the actual distribution of severity is not known. This translates to 7,014 patients having mild glaucoma, 14,028 patients having intermediate glaucoma, and 7,014 patients having severe glaucoma. According to Table 1, the maximal (if everyone adopts the device) direct savings from reduced clinical visits for these patients will be $2,108,197.98 (total cost $4,095,685.02) (Appendix A).

### Appendix A (Maximal Potential Savings Assuming 100% Penetrance)

Using Table 1, we can calculate the total visit cost of all eligible patients:

(113.71 * 7,014) + (139.97 * 14,028) + (190.28 * 7,014) = $4,095,685.02

We can also calculate the maximal potential saving from each group of patients:

Saving from mild glaucoma patients = 42.87 * 7,014 = $300,690.18

Saving from intermediate glaucoma patients = 69.13 * 14,028 = $969,755.64

Saving from severe glaucoma patients = 119.44 * 7,014 = $837,752.16

Total saving = $2,108,197.98

Given that the device has increased benefit for patients with severe glaucoma, we can estimate a penetrance in mild, intermediate, and severe patients of 20%, 40%, and 60% respectively. Again, these percentages are assumptions for the purposes of a hypothetical simulation. This calculates as 1,403 mild cases, 5,611 intermediate cases, and 4,208 severe cases that would undergo implantation of the device, totaling 11,222 individuals (Appendix B).

**Table 1:** **Table 1**: Annual clinic visit costs and savings resulting from continuous intraocular pressure monitoring according to glaucoma severity

*Patient status*		*Visit codes*		*Annual cost per patient*		*Annual saving after implantation per patient*	
Mild/Suspect		1× 92014 ($70.84)		$113.71		$42.87	
		1× 99213 ($42.87)					
Intermediate		1× 92014 ($70.84)		$139.97		$69.13	
		1× 99213 ($42.87)					
		1× 99212 ($26.26)					
Severe		1× 92014 ($70.84)		$190.28		$119.44	
		1× 92012 ($50.31)					
		1× 99213 ($42.87)					
		1× 99212 ($26.26)					

**Table 2:** **Table 2:** Annual savings for patients eligible for intraocular pressure monitoring, assuming all patients have mild, intermediate, or severe glaucoma.

*Glaucoma severity*		*Calculation*		*Total annual saving*	
Mild/Suspect		28,056 patients × $42.87/year/patient		$1,202,760.72/year	
Intermediate		28,056 patients × $69.13/year/patient		$1,939,511.28/year	
Severe		28,056 patients × $119.44/year/patient		$3,351,008.64/year	

### Appendix B (Number of Patients Receiving the Device)

Number of mild patients adopting the device: 20% * 7,014 = 1,403 patients

Number of intermediate patients adopting the device: 40% * 14,028 = 5,611 patients

Number of severe patients adopting the device: 60% * 7,014 = 4,208 patients

Total patients adopting the device: 11,222 patients

We can next determine the cost avoided due to direct visit reduction. The yearly decrease in spending for mild, intermediate, and severe cases would be $60,138.04, $387,902.26, and $502,651.30 respectively. This totals to the annual cost reduction of $950,691.60 for 5% of the Medicare population, a figure which amounts to a 23.21% drop in yearly costs secondary to outpatient appointments (Appendix C).

### Appendix C (Direct Savings Considering Penetrance)

Saving from mild glaucoma patients = 300,690.18 * 20% = $60,138.04

Saving from intermediate glaucoma patients = 969,755.64 * 40% = $387,902.26

Saving from severe glaucoma patients = 837,752.16 * 60% =$502,651.30

Total savings = $950,691.60 which is 23.21% of total cost ($4,095,685.02)

## DISCUSSION

Glaucoma is a debilitating condition affecting millions in the United States and inflicting a financial burden on our healthcare system. Evaluating glaucoma progression relies on continuous, long-term monitoring of IOP as provided by this device. Based on our calculations, this device will likely reduce the financial burden inflicted by glaucoma by decreasing office visits.

The device shows potential in reducing glaucoma’s immense financial burden. As demonstrated in our hypothetical calculation, the device can accomplish a 23.21% decrease in spending related to outpatient appointments. Note that patients with extremely advanced disease (with up to 12 yearly visits) are not included in the analysis. These individuals will likely benefit the most, as additional visits can be saved in more advanced glaucoma. Hence, the actual saving is greater than predicted by the model. One may argue that the device may increase the number of visits, as patients may consult their ophthalmologist every time they discover an elevated IOP. It should be stressed that the device will have an individualized IOP threshold set by the treating physician above which patients should seek medical attention.

On top of quantifiably reduced costs from outpatient visits, the device may also grant further savings due to better medication compliance, reduced loss of productivity, and less traveling. This may be particularly noteworthy if the device is introduced to underserved areas, where patients must travel for extensive distances to get their IOP evaluated. Additionally, due to the advantages of continuous monitoring, it is reasonable to predict that the IOP monitoring device would prevent glaucoma progression to some degree. However, because the device is not approved in America, its role in stabilizing glaucoma is theoretical. Finally, the price of implantation and maintenance would have to be factored into any rigorous cost-effectiveness analysis. For example, according to its manufacturers in Germany, each WIT will probably cost $2,500 initially. After the market stabilizes, the WIT’s price will likely settle at $1,500 each.

The major limitation of this analysis is that because the device is not yet approved in the United States, our analysis must still be validated, as several assumptions have been made. To predict the savings after the implementation of this device, we created a model that takes into account multiple factors and illustrated the model via a hypothetical situation. For the purposes of our model, we assumed that the prevalence of cataract in the nation is similar to the glaucoma population. However, it is known that patients who have undergone a prior penetrating glaucoma surgery are likely to have earlier cataract development. There is no data available on the prevalence of penetrating glaucoma surgery in each of the three severity categories described. Once additional data becomes available, the model can be further revised to predict the device’s financial outlook better.

## CONCLUSION

In conclusion, our model indicates that the implantation of a safe device allowing remote IOP monitoring may alleviate the economic burden imposed on the healthcare system by the current management structure of patients with glaucoma. Further investigations are required to collate the data needed to hypothesize the economic benefits of the system in the Medicare population as well as all other patients requiring glaucoma monitoring.

## CLINICAL SIGNIFICANCE

In an era of elevated healthcare costs, continuous IOP monitoring offers an option to improve the care of glaucoma patients through visit reduction, also resulting in a 23.21% reduction in yearly expenses related to glaucoma clinical visits.
